# Supplementary material for: The effect of generic market entry on antibiotic prescriptions in the United States
Source: Nat Commun. 2021 May 18;12:2937. doi: 10.1038/s41467-021-23049-4 (PMC8131704; doi:10.1038/s41467-021-23049-4)
Supplement: Supplementary file 1 — Supplementary Information [file 41467_2021_23049_MOESM1_ESM.pdf]

The effect of generic market entry on antibiotic prescriptions in the United States

Supplementary information

Antibiotic	Year and Month of first generic approval	Year and Month of first generic prescription	Companies with FDA-approved generics 1 year after generic entry	Companies with FDA-approved generics 2 years after generic entry
Azithromycin	2005 Nov	2005 Nov	5	7
Aztreonam	2010 June	2010 Jul	2	2
Cefdinir	2006 May	2007 May	6	6
Cefpodoxime	2002 May	2004 Jun	2	2
Cefprozil	2005 Nov	2005 Dec	6	7
Cefuroxime axetil	2002 Feb	2002 Mar	3	5
Ciprofloxacin	2004 Mar	2003 May	12	16
Clarithromycin	2004 May	2005 May	10	10
Demeclocycline	2004 Mar	2004 Mar	2	2
Levofloxacin	2010 Dec	2011 Jan	17	20
Meropenem	2010 Jun	2010 Jul	1	3
Ofloxacin	2002 Jan	2003 Sep	4	4
Piperacillin/tazobactam	2009 Sep	2009 Oct	2	4

Supplementary table 1 Generic approvals and initial prescription dates in the United States. Information about generics, including molecule, date of FDA approval, first prescription date in the United States, and number of companies with approved generics 12 and 24 months after generic entry. Note: Under an agreement between Barr pharmaceuticals and Bayer, Barr Pharmaceuticals was granted the right to distribute ciprofloxacin before the end of Bayer's market exclusivity (http://www.cidrap.umn.edu/news-perspective/2004/06/fda-approves-generic-forms-ciprofloxacin)

**Antibiotic**	**6 months**	**12 months**	**18 months**	**24 months**
Aztreonam	181%	406%	2757%	neg value
Cefdinir	-3%	-5%	-8%	-10%
Cefpodoxime	2%	5%	8%	12%
Ciprofloxacin	12%	19%	26%	33%
Ciprofloxacin excluding the anthrax attacks	6%	11%	17%	22%
Levofloxacin	6%	29%	-10%	125%
Ofloxacin	4%	7%	9%	12%

Supplementary table 2 Relative change in level 6, 12, 18, and 24 months after generic entry compared to level sales at the same time points if generic entry would not have taken place.

Antibiotic	Baseline trend			Immediate change in trend		
	Estimate	95% CI	p-value	Estimate	95% CI	p-value
Ciprofloxacin, excluding the anthrax attacks	-2.744	(-15.698, 10.210)	0.675	32.930	(19.497, 46.362)	<0.001
Ciprofloxacin, removing outlier	-8.208	(-11.925, -4.491)	<0.001	37.677	(32.705, 42.650	<0.001
Meropenem, removing beginning of time series	0.002	(0.001, 0.003)	<0.001	0.001	(-0.002, 0.004)	0.607
Piperacillin/tazobactam, removing beginning of time series	-0.003	(-0.006, -0.000)	0.033	0.002	(-0.002, 0.006)	0.276
Aztreonam, removing shortage	-0.001	(-0.002-0.001)	<0.001	0.001	(-0.001, 0.004)	0.375

Supplementary table 3 Sensitivity analysis, trends before and after generic entry. Ciprofloxacin: restricting time series to October 2001 to January 2010, thereby excluding the anthrax attacks. Ciprofloxacin: removing outlier. Meropenem: removing data before 2005. Piperacillin/tazobactam; removing data before 2007. Aztreonam: removing data from June 2009 to June 2010. Results of the interrupted time series analysis, measuring the changes in number of antibiotic prescriptions per one million population per month. The column labeled baseline trend corresponds to the trend in use before generic entry. The column labeled immediate change in trend corresponds to the change in trend after generic entry. Either segmented regression was used with Prais-Winsten regression when autocorrelation was present. Two-sided test was used with no adjustment for multiple comparisons.

Antibiotic	Change in level 6 months after generic introduction			Change in level 12 months after generic introduction			Change in level 18 months after generic introduction			Change in level 24 months after generic introduction		
	Estimate	95% CI	p-value	Estimate	95% CI	p-value	Estimate	95% CI	p-value	Estimate	95% CI	p-value
Ciprofloxacin, excluding the anthrax attacks	211.021	(-75.994, 498.036)	0.148	408.600	(59.543, 757.656)	0.022	606.178	(188.693, 1023.663)	0.005	803.756	(314.126, 1293.386)	0.002
Ciprofloxacin, removing outlier	453.246	(266.756, 639.737)	<0.001	679.310	(483.996 , 874.624)	<0.001	905.374	(697.295, 1113.453)	<0.001	1131.438	(907.326, 1355.55)	<0.001
Meropenem, removing beginning of time series	-0.052	(-0.105, 0.002)	0.057	-0.047	(-0.099, 0.005)	0.074	-0.042	(-0.099, 0.014)	0.141	-0.037	(-0.103, 0.029)	0.266
Piperacillin/tazobactam, removing beginning of time series	-0.033	(-0.113, 0.048)	0.423	-0.020	(-0.112, 0.071)	0.657	-0.008	(-0.114, 0.098)	0.877	0.004	(-0.119, 0.127)	0.949
Aztreonam, removing shortage	0.027	(-0.017, 0.072)	0.232	0.034	(-0.005, 0.073)	0.086	0.041	(0.001, 0.081)	0.042	0.049	(0.002, 0.095)	0.041

Supplementary table 4 Sensitivity analysis, level change over time. Ciprofloxacin: restricting time series to October 2001 to January 2010, thereby excluding the anthrax attacks. Ciprofloxacin: removing outlier. Meropenem: removing data before 2005. Piperacillin/tazobactam; removing data before 2007. Aztreonam: removing data from June 2009 to June 2010. Results of the interrupted time series analysis, measuring the change in number of antibiotic prescriptions per one million population 6, 12, 18, and 24 months after generic entry. Either segmented regression was used with Prais-Winsten regression when autocorrelation was present. Two-sided test was used with no adjustment for multiple comparisons.

Antibiotic	Original brand name	Company	FDA approval date	Class	Form	Indications according to latest available FDA label
Azithromycin	Zithromax	Pfizer	1991 Nov	Macrolide	IV, PO	IV: sexually transmitted diseases, mycobacterial infections. PO: community-acquired pneumonia in adults, pelvic inflammatory disease.
Aztreonam	Azactam	Bristol Myers Squibb	1986 Dec	Monobactam	IV	Urinary tract infections (complicated and uncomplicated), lower respiratory tract infection, septicaemia, skin and skin-structure infections, intra-abdominal infections, gynaecologic infections.
Cefdinir	Omnicef	Abbvie	1997 Dec	3rd generation cephalosporin	PO	Adults and adolescents: community-acquired pneumonia, acute exacerbations of chronic bronchitis, acute maxillary sinusitis, pharyngitis/tonsillitis, uncomplicated skin and skin structure infections. Paediatric patients: acute bacterial otitis media, pharyngitis/tonsillitis, uncomplicated skin and skin structure infections.
Cefpodoxime	Vantin	Pharmacia and Up John	1992 Aug	3rd generation cephalosporin	PO	Pharyngitis and/or tonsillitis, community-acquired pneumonia, acute bacterial exacerbation of chronic bronchitis, acute uncomplicated urethral and cervical gonorrhoea, acute uncomplicated ano-rectal infections in women, uncomplicated skin and skin structure infections, acute maxillary sinusitis, uncomplicated urinary tract infections (cystitis).
Cefprozil	Cefzil	Cordon Pharma	1991 Dec	2nd generation cephalosporin	PO	Pharyngitis/tonsillitis, otitis media, acute sinusitis, acute bacterial exacerbation of chronic bronchitis, uncomplicated skin and skin-structure infections.
Cefuroxime axetil	Ceftin	Glaxo Smith Kline	1987 Dec	2nd generation cephalosporin	PO	Adults and paediatric patients: pharyngitis/tonsillitis, acute bacterial otitis media, acute bacterial maxillary sinusitis. Adults and paediatric patients 13 years and older: acute bacterial exacerbations of chronic bronchitis, uncomplicated skin and skin-structure infections, uncomplicated urinary tract infections, uncomplicated gonorrhoea, early Lyme disease. Paediatric patients: impetigo.
Ciprofloxacin	Cipro	Bayer Healthcare	1987 Oct	Fluoroquinolones	IV, PO	Skin and skin structure infections, bone and joint infections, complicated intra-abdominal infections, infectious diarrhoea, typhoid fever (enteric fever), uncomplicated cervical and urethral gonorrhoea, inhalational anthrax post-exposure, plague, chronic bacterial prostatitis, lower respiratory tract infections, acute exacerbation of chronic bronchitis, urinary tract infections, urinary tract infections (UTI), acute uncomplicated cystitis, complicated UTI and pyelonephritis, acute sinusitis.

Clarithromycin	Biaxin	Abbvie	1991 Oct	Macrolide	PO	Acute bacterial exacerbation of chronic bronchitis, acute maxillary sinusitis, community-acquired pneumonia, pharyngitis/tonsillitis, uncomplicated skin and skin structure infections, acute otitis media in paediatric patients, treatment and prophylaxis of disseminated mycobacterial infections, helicobacter pylori infection and duodenal ulcer disease.
Demeclocycline	Declomycin	Lederle	1960 Nov	Tetracycline	PO	Rocky Mountain spotted fever, typhus fever and the typhus group, Q fever, rickettsial pox, and tick fevers caused by rickettsia, respiratory tract infections, lymphogranuloma venereum, psittacosis, trachoma, inclusion conjunctivitis, non-gonococcal urethritis in adults, chancroid, plague, tularaemia, cholera, brucellosis, granuloma inguinale, urinary tract infections, syphilis, listeriosis, anthrax.
Levofloxacin	Levaquin	Janssen Pharma	1996 Dec	Fluoroquinolones	IV, PO	Pneumonia (nosocomial and community acquired), skin and skin structure infections (complicated and uncomplicated), chronic bacterial prostatitis, inhalational, anthrax incl. post-exposure, plague, urinary tract infections (complicated and uncomplicated), acute pyelonephritis, acute bacterial exacerbation of chronic bronchitis, acute bacterial sinusitis.
Meropenem	Merrem	Pfizer	1996 Jun	Carbapenems	IV	Adults and paediatric patients: complicated skin and skin structure infections, complicated intra-abdominal infections, bacterial meningitis.
Ofloxacin	Floxin	Janssen Pharma	1990 Dec	Fluoroquinolones	IV, PO	Acute bacterial exacerbations of chronic bronchitis, community-acquired pneumonia, uncomplicated skin and skin structure infections, acute uncomplicated urethral and cervical gonorrhoea, non-gonococcal urethritis and cervicitis, mixed infections of the urethra and cervix, acute pelvic inflammatory disease.
Piperacillin/ tazobactam	Zosyn	Wyeth Pharma	1993 Oct	Combination of penicillin and beta-lactamase inhibitor	IV	Intra-abdominal infections, skin and skin structure infections, female pelvic infections, community-acquired pneumonia, nosocomial pneumonia.

Supplementary table 5 Antibiotics and original brand approval dates in the United States. Information about researched antibiotics, including molecule and original brand name, company responsible for development and approval, date of FDA approval, formulation, and indication according to FDA approval.

Antibiotic	Analysis
Azithromycin	Prais-Winsten and adjusted for seasonality
Aztreonam	Prais-Winsten
Cefdinir	Prais-Winsten, log-transformation and adjusted for seasonality
Cefpodoxime	Prais-Winsten, log-transformation and adjusted for seasonality
Cefprozil	Prais-Winsten, log-transformation and adjusted for seasonality
Cefuroxime axetil	Prais-Winsten and adjusted for seasonality
Ciprofloxacin	Prais-Winsten
Clarithromycin	Prais-Winsten and adjusted for seasonality
Demeclocycline	Prais-Winsten and adjusted for seasonality
Levofloxacin	Quadratic segmented regression and adjusted for seasonality
Meropenem	Prais-Winsten
Ofloxacin	Prais-Winsten and log-transformation
Piperacillin/tazobactam	Prais-Winsten

Supplementary table 6 Analysis methods.

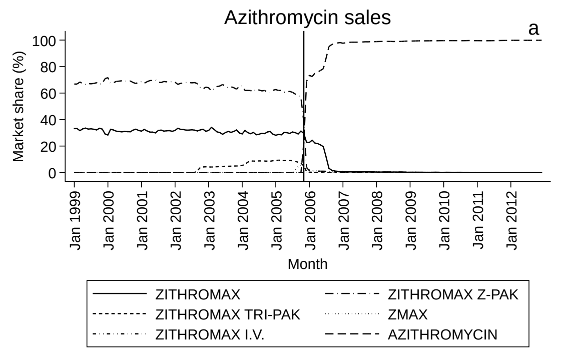

Brand: ZITHROMAX, ZITHROMAX Z-PAK, ZITHROMAX TRI-PAK, ZMAX, ZITHROMAX I.V.
Generic: AZITHROMYCIN
Generic entry: November 2005

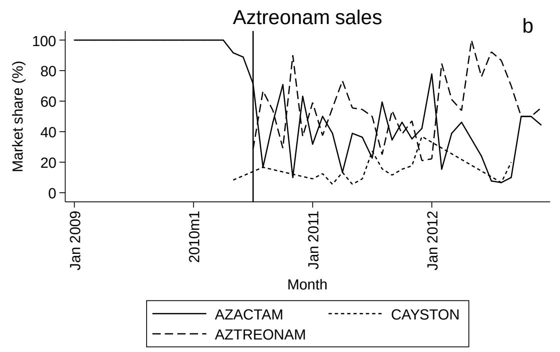

Brand: Azactam, Generic: Aztreonam
Generic entry: July 2010
(Cayston is a brand product kept to visualize consumption but was not included in analysis)

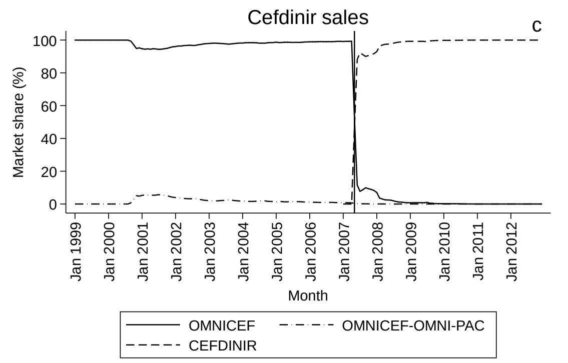

Brand: Omnicef, Omnicef-Omni-Pac, Generic: Cefdinir
Generic entry: May 2007

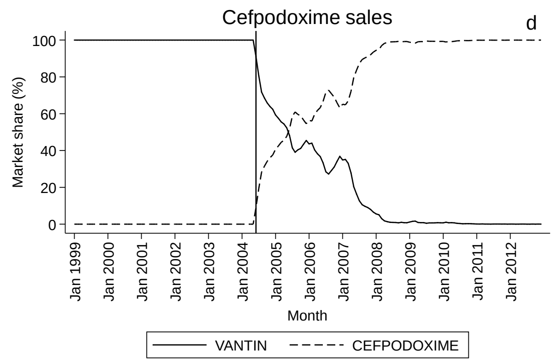

Brand: Vantin, Generic: Cefpodoxime
Generic entry: June 2004

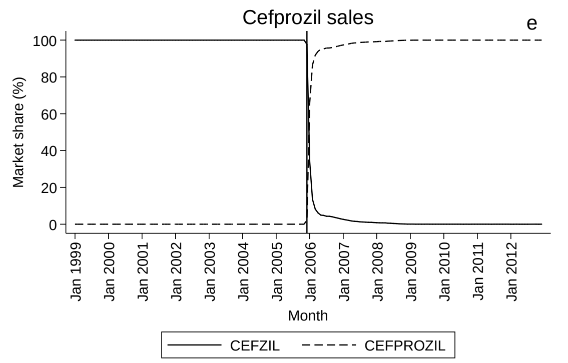

Brand: Cefzil, Generic: Cefprozil
Generic entry: December 2005

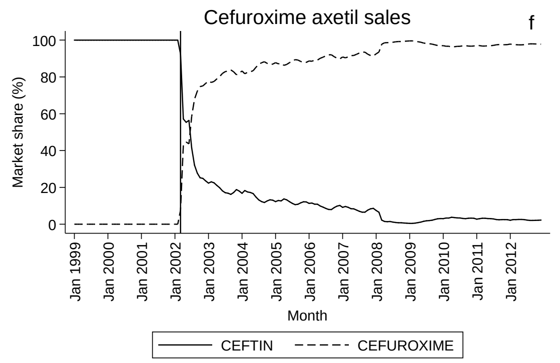

Brand: Ceftin, Generic: Cefuroxime
Generic entry: March 2002

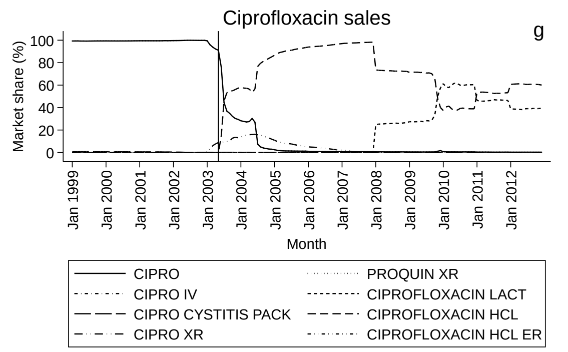

Brand: Cipro, Cipro IV, Cipro Cystitis Pack, Cipro XR
Generic: Ciprofloxacin Lact, Ciprofloxacin HCL, Ciprofloxacin HCL ER, Proquin XR
Generic entry: May 2003

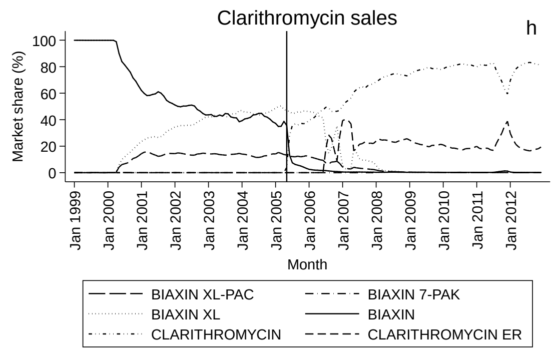

Brand: Biaxin, Biaxin XL, Biaxin XL-Pac, Biaxin 7-Pac
Generic: Clarithromycin, Clarithromycin ER
Generic entry: June 2006

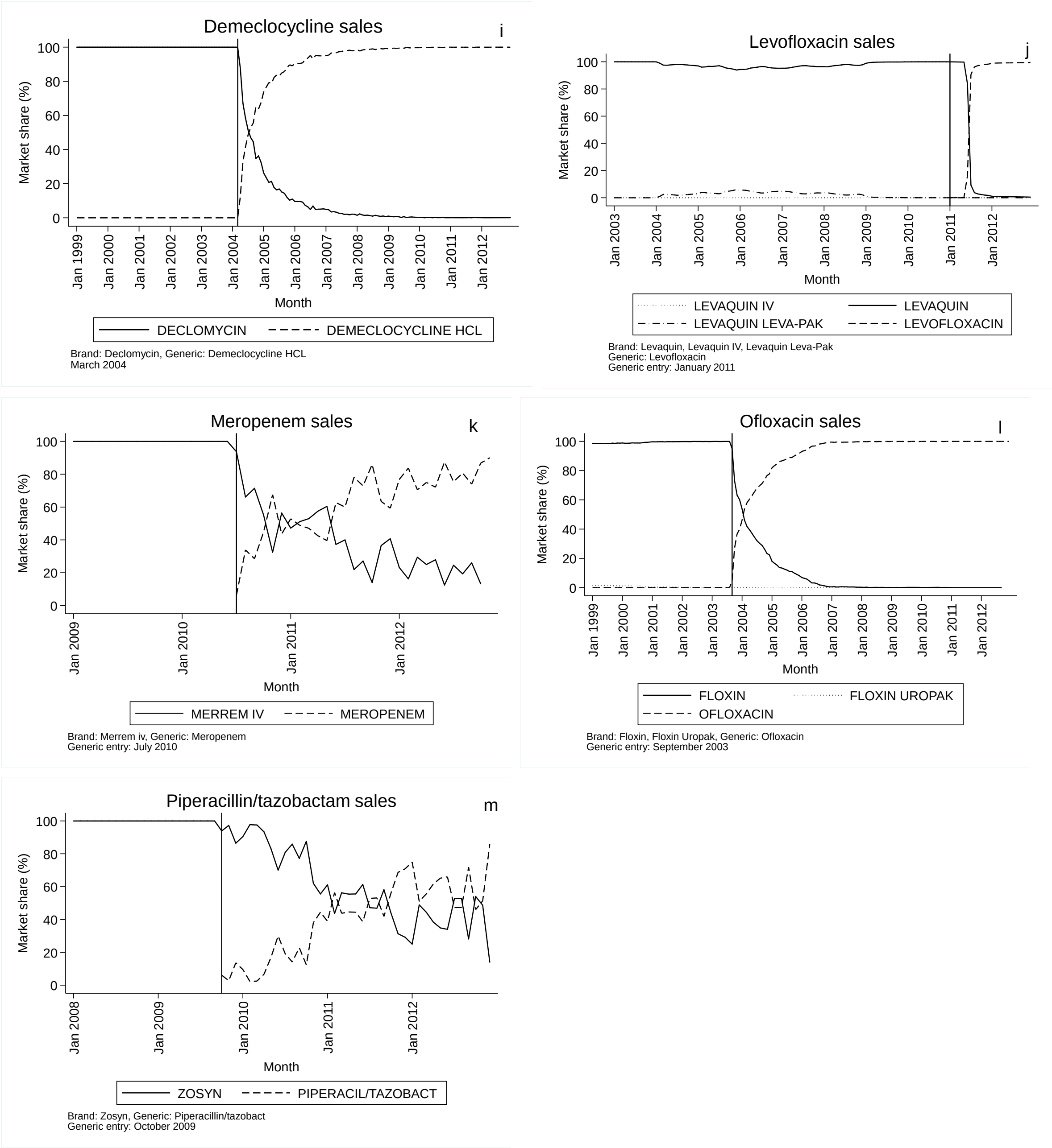

Supplementary figure 1 Markets for brand and generic products. Level of prescriptions, for brand and generic products, including month of generic entry a) Azithromycin. b) Aztreonam. c) Cefdinir. d) Cefpodoxime. e) Cefprozil. f) Cefuroxime axetil. g) Ciprofloxacin. h) Clarithromycin. i) Demeclocycline. j) Levofloxacin. k) Meropenem. l) Ofloxacin. m) Piperacillin/tazobactam.

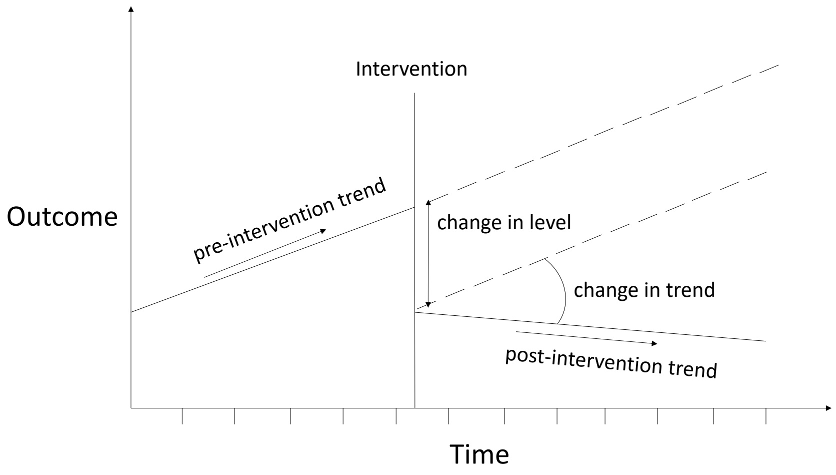

Supplementary figure 2 Schematic figure of ITS effect estimates.

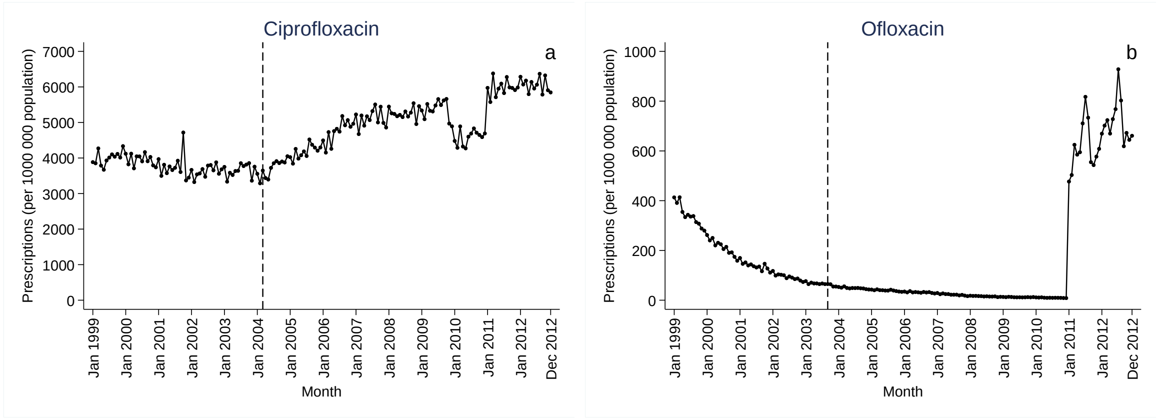

Supplementary figure 3 a) Full time series for ciprofloxacin. b) Full time series for ofloxacin.

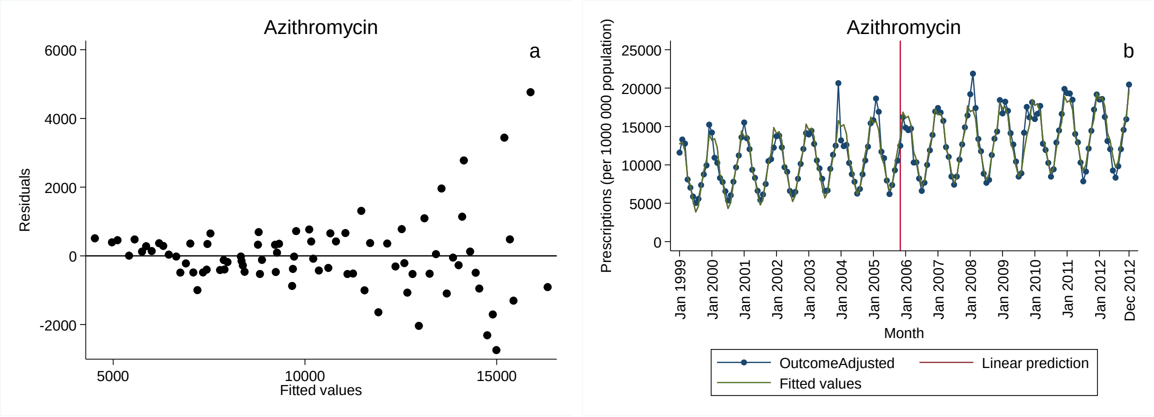

c

```
Akaike's information criterion and Bayesian information criterion
```

Model	Obs	ll(null)	ll(model)	df	AIC	BIC
.	168	-1628.581	-1413.833	15	2857.666	2904.526

Note: N=Obs used in calculating BIC; see **[R] BIC note**.

d

```
Akaike's information criterion and Bayesian information criterion
```

Model	Obs	ll(null)	ll(model)	df	AIC	BIC
.	168	.	-1398.819	15	2827.638	2874.498

Note: N=Obs used in calculating BIC; see **[R] BIC note**.

Supplementary figure 4 Azithromycin. a) Graph to assess linearity. b) Visual inspection of linear regression model and Prais-Winsten model to compare best fit. c) Akaike information criterion and the Bayesian information criterion for linear model. d) Akaike information criterion and the Bayesian information criterion for Prais-Winsten model.

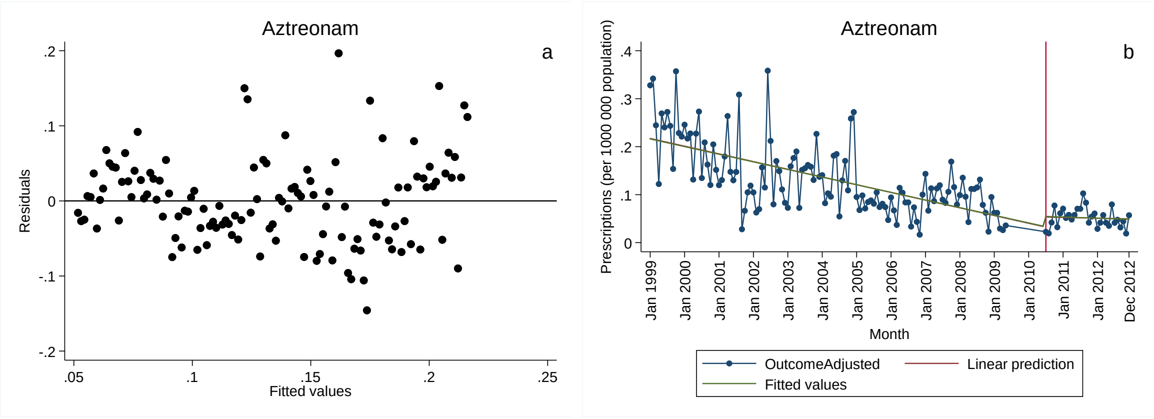

c

Akaike's information criterion and Bayesian information criterion

Model	Obs	ll(null)	ll(model)	df	AIC	BIC
.	166	191.1581	258.9512	4	-509.9025	-497.4545

Note: N=Obs used in calculating BIC; see **[R] BIC note**.

d

Akaike's information criterion and Bayesian information criterion

Model	Obs	ll(null)	ll(model)	df	AIC	BIC
.	166	.	263.0227	4	-518.0454	-505.5975

Note: N=Obs used in calculating BIC; see **[R] BIC note**.

Supplementary figure 5 Aztreonam. a) Graph to assess linearity. b) Visual inspection of linear regression model and Prais-Winsten model to compare best fit. c) Akaike information criterion and the Bayesian information criterion for linear model. d) Akaike information criterion and the Bayesian information criterion for Prais-Winsten model.

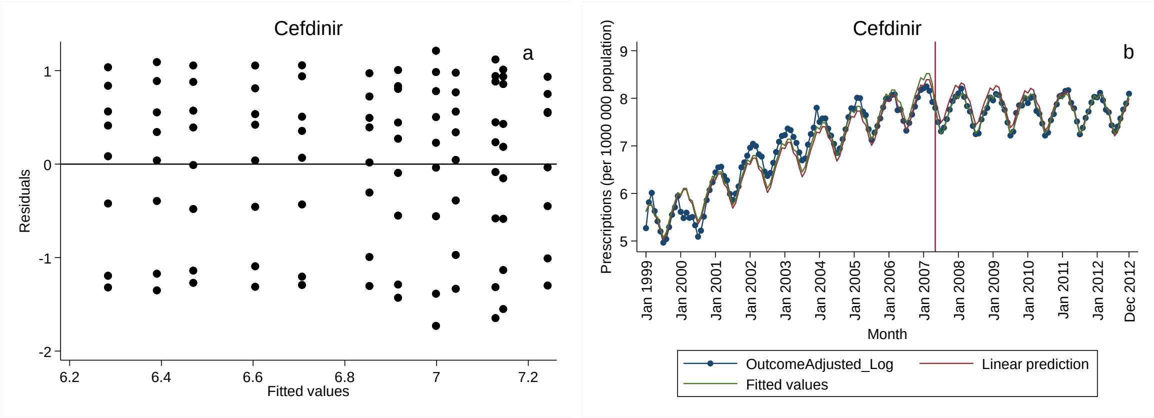

c

Akaike's information criterion and Bayesian information criterion

Model	Obs	ll(null)	ll(model)	df	AIC	BIC
.	168	-210.8235	69.4899	15	-108.9798	-62.12033

Note: N=Obs used in calculating BIC; see **[R] BIC note**.

d

Akaike's information criterion and Bayesian information criterion

Model	Obs	ll(null)	ll(model)	df	AIC	BIC
.	168	.	182.5845	15	-335.1689	-288.3094

Note: N=Obs used in calculating BIC; see **[R] BIC note**.

Supplementary figure 6 Cefdinir. a) Graph to assess linearity. b) Visual inspection of linear regression model and Prais-Winsten model to compare best fit. c) Akaike information criterion and the Bayesian information criterion for linear model. d) Akaike information criterion and the Bayesian information criterion for Prais-Winsten model.

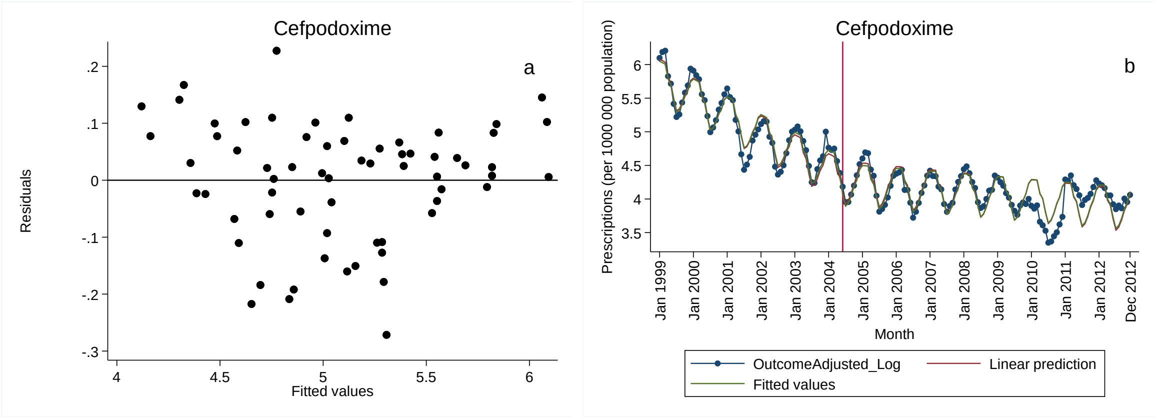

c

Akaike's information criterion and Bayesian information criterion

Model	Obs	ll(null)	ll(model)	df	AIC	BIC
.	168	-158.921	75.59265	15	-121.1853	-74.32583

Note: N=Obs used in calculating BIC; see **[R] BIC note**.

d

Akaike's information criterion and Bayesian information criterion

Model	Obs	ll(null)	ll(model)	df	AIC	BIC
.	168	.	196.724	15	-363.448	-316.5886

Note: N=Obs used in calculating BIC; see **[R] BIC note**.

Supplementary figure 7 Cefpodoxime. a) Graph to assess linearity. b) Visual inspection of linear regression model and Prais-Winsten model to compare best fit. c) Akaike information criterion and the Bayesian information criterion for linear model. d) Akaike information criterion and the Bayesian information criterion for Prais-Winsten model.

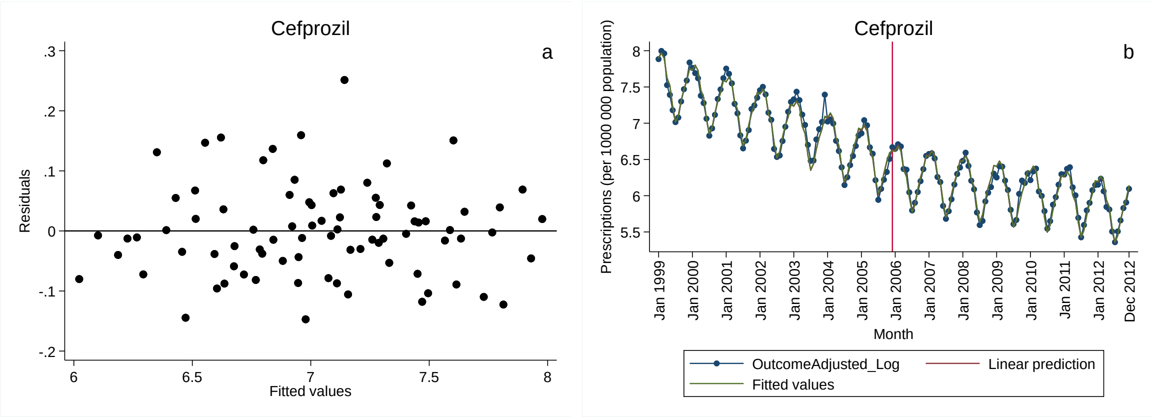

c

Akaike's information criterion and Bayesian information criterion

Model	Obs	ll(null)	ll(model)	df	AIC	BIC
.	168	-158.8696	206.4411	15	-382.8822	-336.0228

Note: N=Obs used in calculating BIC; see **[R] BIC note**.

d

Akaike's information criterion and Bayesian information criterion

Model	Obs	ll(null)	ll(model)	df	AIC	BIC
.	168	.	238.5922	15	-447.1844	-400.3249

Note: N=Obs used in calculating BIC; see **[R] BIC note**.

Supplementary figure 8 Cefprozil. a) Graph to assess linearity. b) Visual inspection of linear regression model and Prais-Winsten model to compare best fit. c) Akaike information criterion and the Bayesian information criterion for linear model. d) Akaike information criterion and the Bayesian information criterion for Prais-Winsten model.

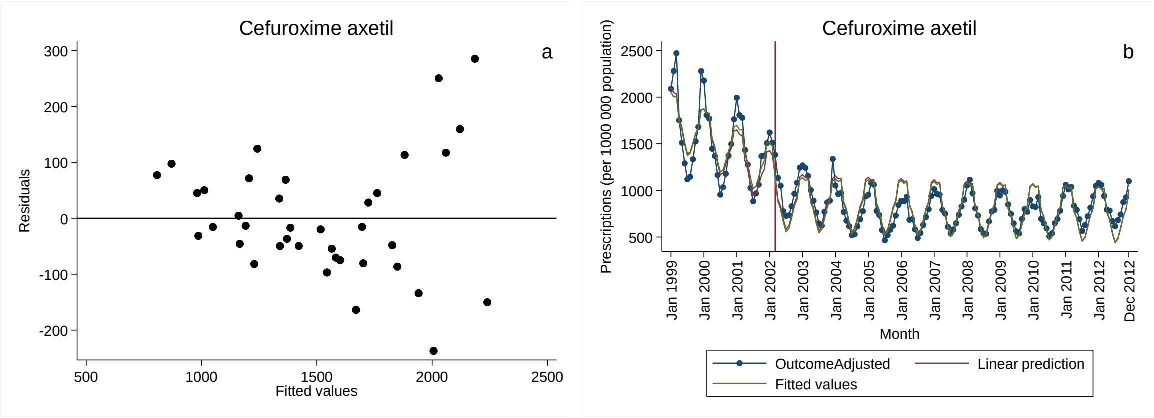

c

Akaike's information criterion and Bayesian information criterion

Model	Obs	ll(null)	ll(model)	df	AIC	BIC
.	168	-1240.652	-1049.937	15	2129.875	2176.734

Note: N=Obs used in calculating BIC; see **[R] BIC note**.

d

Akaike's information criterion and Bayesian information criterion

Model	Obs	ll(null)	ll(model)	df	AIC	BIC
.	168	.	-984.6145	15	1999.229	2046.089

Note: N=Obs used in calculating BIC; see **[R] BIC note**.

Supplementary figure 9 Cefuroxime axetil. a) Graph to assess linearity. b) Visual inspection of linear regression model and Prais-Winsten model to compare best fit. c) Akaike information criterion and the Bayesian information criterion for linear model. d) Akaike information criterion and the Bayesian information criterion for Prais-Winsten model.

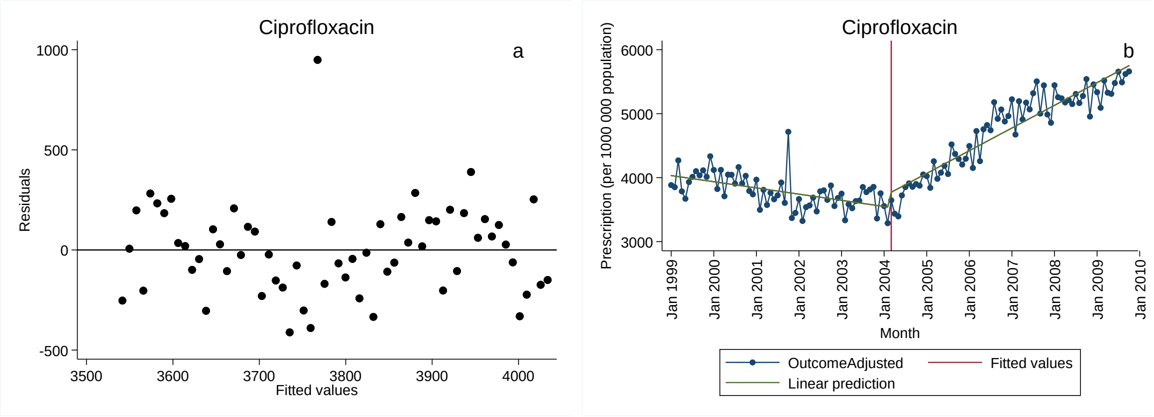

c

Akaike's information criterion and Bayesian information criterion

Model	Obs	ll(null)	ll(model)	df	AIC	BIC
.	130	-1033.798	-888.2704	4	1784.541	1796.011

Note: N=Obs used in calculating BIC; see **[R] BIC note**.

d

Akaike's information criterion and Bayesian information criterion

Model	Obs	ll(null)	ll(model)	df	AIC	BIC
.	130	.	-887.3561	4	1782.712	1794.182

Note: N=Obs used in calculating BIC; see **[R] BIC note**.

Supplementary figure 10 Ciprofloxacin. a) Graph to assess linearity. b) Visual inspection of linear regression model and Prais-Winsten model to compare best fit. c) Akaike information criterion and the Bayesian information criterion for linear model. d) Akaike information criterion and the Bayesian information criterion for Prais-Winsten model.

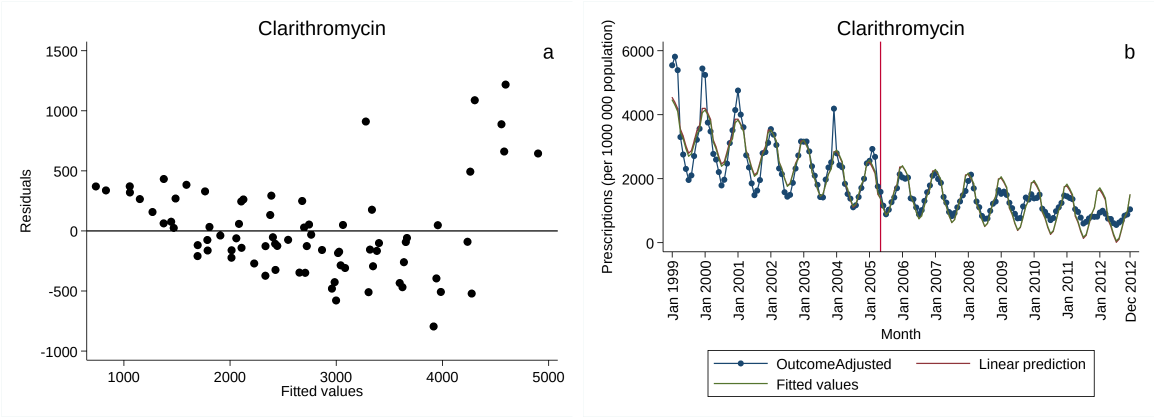

c

Akaike's information criterion and Bayesian information criterion

Model	Obs	ll(null)	ll(model)	df	AIC	BIC
.	168	-1410.987	-1236.212	15	2502.424	2549.283

Note: N=Obs used in calculating BIC; see **[R] BIC note**.

d

Akaike's information criterion and Bayesian information criterion

Model	Obs	ll(null)	ll(model)	df	AIC	BIC
.	168	.	-1184.284	15	2398.567	2445.427

Note: N=Obs used in calculating BIC; see **[R] BIC note**.

Supplementary figure 11 Clarithromycin. a) Graph to assess linearity. b) Visual inspection of linear regression model and Prais-Winsten model to compare best fit. c) Akaike information criterion and the Bayesian information criterion for linear model. d) Akaike information criterion and the Bayesian information criterion for Prais-Winsten model.

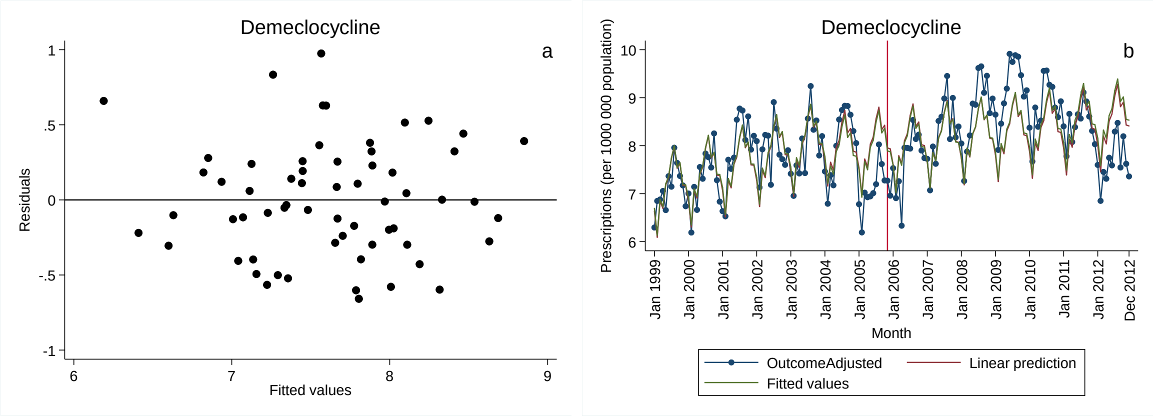

c

Akaike's information criterion and Bayesian information criterion

Model	Obs	ll(null)	ll(model)	df	AIC	BIC
.	168	-207.3962	-139.5829	15	309.1658	356.0252

Note: N=Obs used in calculating BIC; see **[R] BIC note**.

d

Akaike's information criterion and Bayesian information criterion

Model	Obs	ll(null)	ll(model)	df	AIC	BIC
.	168	.	-78.19204	15	186.3841	233.2435

Note: N=Obs used in calculating BIC; see **[R] BIC note**.

Supplementary figure 12 Demeclocycline. a) Graph to assess linearity. b) Visual inspection of linear regression model and Prais-Winsten model to compare best fit. c) Akaike information criterion and the Bayesian information criterion for linear model. d) Akaike information criterion and the Bayesian information criterion for Prais-Winsten model.

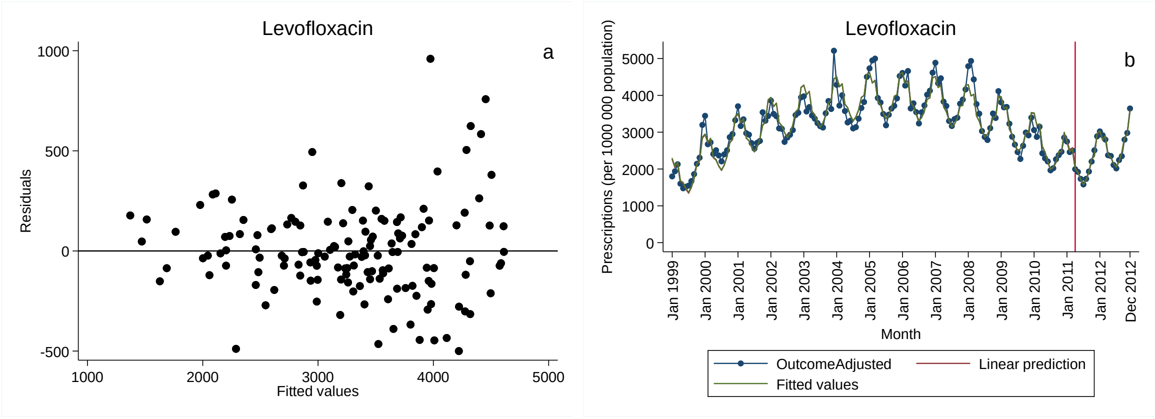

c

Akaike's information criterion and Bayesian information criterion

Model	Obs	ll(null)	ll(model)	df	AIC	BIC
.	168	-1363.88	-1138.753	17	2311.506	2364.613

Note: N=Obs used in calculating BIC; see **[R] BIC note**.

d

Akaike's information criterion and Bayesian information criterion

Model	Obs	ll(null)	ll(model)	df	AIC	BIC
.	168	.	-1116.241	17	2266.482	2319.589

Note: N=Obs used in calculating BIC; see **[R] BIC note**.

Supplementary figure 13 Levofloxacin. a) Graph to assess linearity. b) Visual inspection of linear regression model and Prais-Winsten model to compare best fit. c). Akaike information criterion and the Bayesian information criterion for linear model. d) Akaike information criterion and the Bayesian information criterion for Prais-Winsten model.

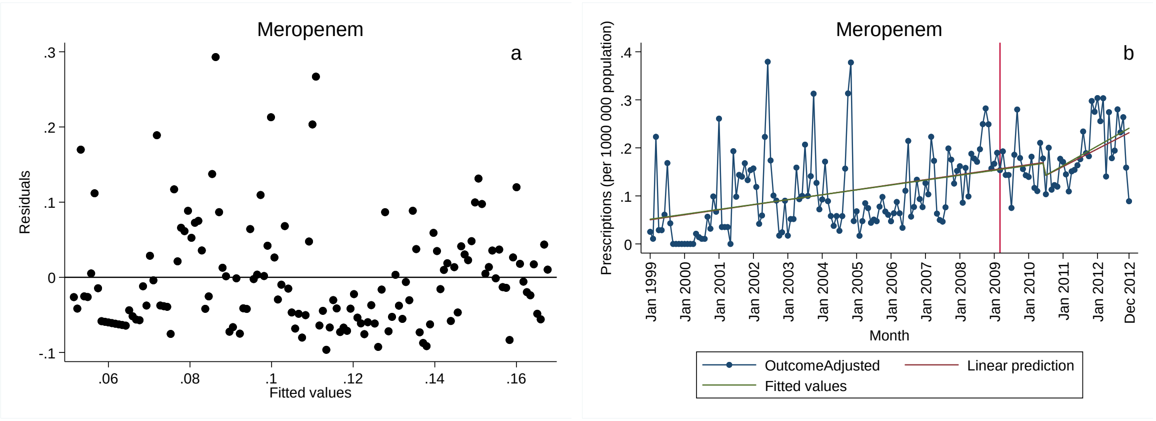

c

Akaike's information criterion and Bayesian information criterion

Model	Obs	ll(null)	ll(model)	df	AIC	BIC
.	152	167.9774	188.3585	4	-368.7171	-356.6215

Note: N=Obs used in calculating BIC; see **[R] BIC note**.

d

Akaike's information criterion and Bayesian information criterion

Model	Obs	ll(null)	ll(model)	df	AIC	BIC
.	152	.	201.531	4	-395.0621	-382.9666

Note: N=Obs used in calculating BIC; see **[R] BIC note**.

Supplementary figure 14 Meropenem. a) Graph to assess linearity. b) Visual inspection of linear regression model and Prais-Winsten model to compare best fit. c) Akaike information criterion and the Bayesian information criterion for linear model. d) Akaike information criterion and the Bayesian information criterion for Prais-Winsten model.

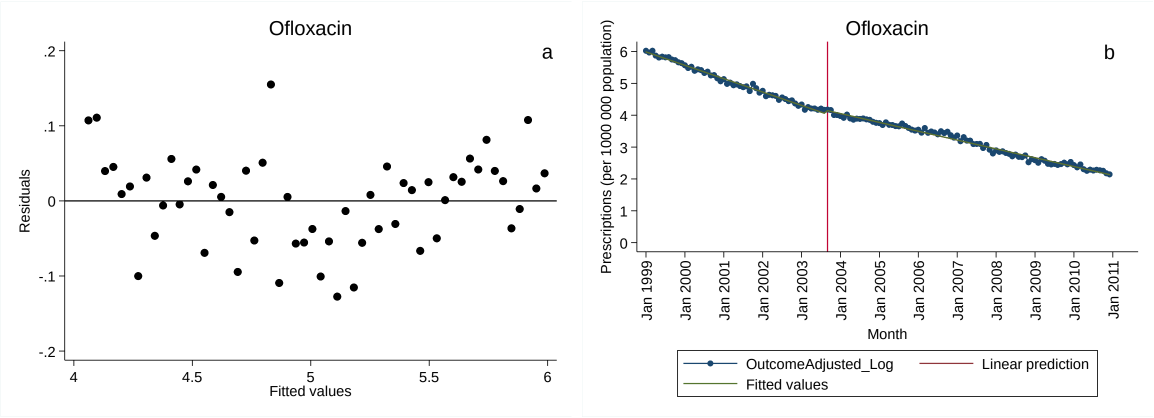

c

Akaike's information criterion and Bayesian information criterion

Model	Obs	ll(null)	ll(model)	df	AIC	BIC
.	144	-216.4508	179.7673	4	-351.5346	-339.6553

Note: N=Obs used in calculating BIC; see **[R] BIC note**.

d

Akaike's information criterion and Bayesian information criterion

Model	Obs	ll(null)	ll(model)	df	AIC	BIC
.	144	.	193.1208	4	-378.2416	-366.3624

Note: N=Obs used in calculating BIC; see **[R] BIC note**.

Supplementary figure 15 Ofloxacin. a) Graph to assess linearity. b) Visual inspection of linear regression model and Prais-Winsten model to compare best fit. c) Akaike information criterion and the Bayesian information criterion for linear model. d) Akaike information criterion and the Bayesian information criterion for Prais-Winsten model.

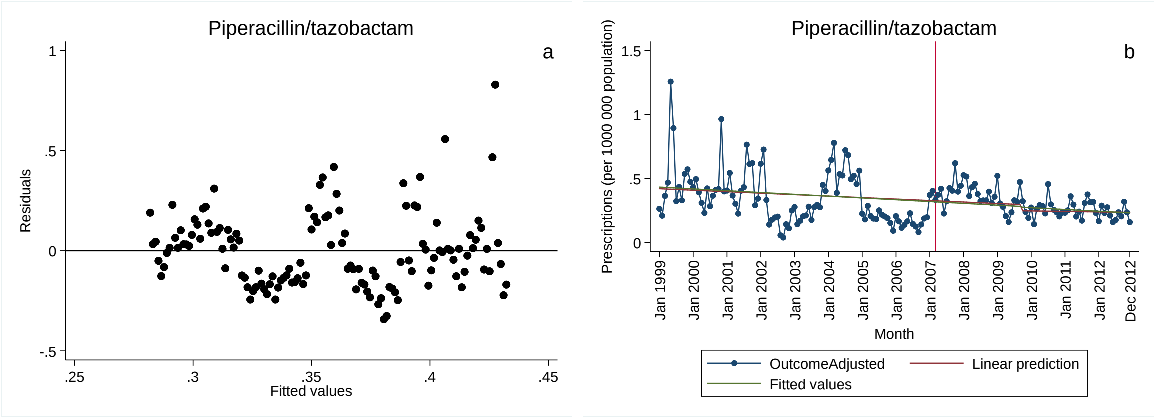

c

Akaike's information criterion and Bayesian information criterion

Model	Obs	ll(null)	ll(model)	df	AIC	BIC
.	168	53.32874	63.21276	4	-118.4255	-105.9297

Note: N=Obs used in calculating BIC; see **[R] BIC note**.

d

Akaike's information criterion and Bayesian information criterion

Model	Obs	ll(null)	ll(model)	df	AIC	BIC
.	168	.	98.79636	4	-189.5927	-177.0969

Note: N=Obs used in calculating BIC; see **[R] BIC note**.

Supplementary figure 16 Piperacillin/tazobactam. a) Graph to assess linearity. b) Visual inspection of linear regression model and Prais-Winsten model to compare best fit. c) Akaike information criterion and the Bayesian information criterion for linear model. d) Akaike information criterion and the Bayesian information criterion for Prais-Winsten model.
