# Supplementary material for: The effect of generic market entry on antibiotic prescriptions in the United States
Source: Nat Commun. 2021 May 18;12:2937. doi: 10.1038/s41467-021-23049-4 (PMC8131704; doi:10.1038/s41467-021-23049-4)
Supplement: Supplementary file 3 — Reporting Summary [file 41467_2021_23049_MOESM3_ESM.pdf]

nature research

Corresponding author(s): Cecilia Kållberg

Last updated by author(s): Mar 21, 2021

# Reporting Summary

Nature Research wishes to improve the reproducibility of the work that we publish. This form provides structure for consistency and transparency in reporting. For further information on Nature Research policies, see our Editorial Policies and the Editorial Policy Checklist.

## Statistics

For all statistical analyses, confirm that the following items are present in the figure legend, table legend, main text, or Methods section.

| n/a | Confirmed | |
|---|---|---|
| ☐ | ☒ | The exact sample size ($n$) for each experimental group/condition, given as a discrete number and unit of measurement |
| ☐ | ☒ | A statement on whether measurements were taken from distinct samples or whether the same sample was measured repeatedly |
| ☐ | ☒ | The statistical test(s) used AND whether they are one- or two-sided<br>*Only common tests should be described solely by name; describe more complex techniques in the Methods section.* |
| ☒ | ☐ | A description of all covariates tested |
| ☐ | ☒ | A description of any assumptions or corrections, such as tests of normality and adjustment for multiple comparisons |
| ☐ | ☒ | A full description of the statistical parameters including central tendency (e.g. means) or other basic estimates (e.g. regression coefficient) AND variation (e.g. standard deviation) or associated estimates of uncertainty (e.g. confidence intervals) |
| ☐ | ☒ | For null hypothesis testing, the test statistic (e.g. $F$, $t$, $r$) with confidence intervals, effect sizes, degrees of freedom and $P$ value noted<br>*Give P values as exact values whenever suitable.* |
| ☒ | ☐ | For Bayesian analysis, information on the choice of priors and Markov chain Monte Carlo settings |
| ☒ | ☐ | For hierarchical and complex designs, identification of the appropriate level for tests and full reporting of outcomes |
| ☒ | ☐ | Estimates of effect sizes (e.g. Cohen's $d$, Pearson's $r$), indicating how they were calculated |

*Our web collection on statistics for biologists contains articles on many of the points above.*

## Software and code

Policy information about availability of computer code

| | |
|---|---|
| Data collection | No software was used for data Data was obtained from the IQVIA Xponent database, owned by IQVIA. |
| Data analysis | We conducted interrupted time series analysis using STATA15. All codes that were written as part of the research will be available. |

For manuscripts utilizing custom algorithms or software that are central to the research but not yet described in published literature, software must be made available to editors and reviewers. We strongly encourage code deposition in a community repository (e.g. GitHub). See the Nature Research guidelines for submitting code & software for further information.

## Data

Policy information about availability of data

All manuscripts must include a data availability statement. This statement should provide the following information, where applicable:

- Accession codes, unique identifiers, or web links for publicly available datasets
- A list of figures that have associated raw data
- A description of any restrictions on data availability

The data that support the findings of this study are available from IQVIA (www.iqvia.com), but restrictions apply to the availability of these data, which were used under license for the current study, and so are not publically available. The terms of IQVIA’s licensing agreement preclude sharing of the data. Other researchers may purchase the data from IQVIA directly.

# Field-specific reporting

Please select the one below that is the best fit for your research. If you are not sure, read the appropriate sections before making your selection.

☐ Life sciences ☒ Behavioural & social sciences ☐ Ecological, evolutionary & environmental sciences

For a reference copy of the document with all sections, see nature.com/documents/nr-reporting-summary-flat.pdf

# Behavioural & social sciences study design

All studies must disclose on these points even when the disclosure is negative.

| | |
|---|---|
| Study description | In this quantitative study we used interrupted time series analysis to analyse the impact of generic entry on antibiotic sales in the US. We measured total sales of 13 different antibiotics before and after the introduction of generic version. Effect of generic entry was measured 6, 12, 18 and 24 months after generic entry. |
| Research sample | We analysed data for 13 different antibiotic, including both originals and generics for each antibiotic, to estimate the impact on overall sales when generics are introduced to market. The selection of the 13 antibiotics were made based on specific inclusion criteria (see Sampling strategy below). Data on antibiotic sales for the 13 antibiotics was obtained from IQVIA Xponent (see Data above). |
| Sampling strategy | Antibiotics were selected based on the following inclusion criteria: antibiotics for systemic use for which a generic product entered the market between 2000 and 2012 of the same formulation and strength based on NDA/ANDA-status, obtained from the FDA website (https://www.accessdata.fda.gov/scripts/cder/daf/index.cfm). 13 antibiotics filled the inclusion criteria. |
| Data collection | Data on antibiotic sales was obtained from the IQVIA Xponent database through a contract between IQVIA and Center for Disease Dynamics, Economics and Policy (CDDEP). |
| Timing | We included data on antibiotic sales between January 2000 and December 2012. |
| Data exclusions | After identifying antibiotics that met the inclusion criteria, no data was excluded. |
| Non-participation | n/a |
| Randomization | n/a. Since our study was not a controlled experiment we were not able to randomize. |

# Reporting for specific materials, systems and methods

We require information from authors about some types of materials, experimental systems and methods used in many studies. Here, indicate whether each material, system or method listed is relevant to your study. If you are not sure if a list item applies to your research, read the appropriate section before selecting a response.

## Materials & experimental systems

| n/a | Involved in the study | |
|---|---|---|
| ☒ | ☐ | Antibodies |
| ☒ | ☐ | Eukaryotic cell lines |
| ☒ | ☐ | Palaeontology and archaeology |
| ☒ | ☐ | Animals and other organisms |
| ☒ | ☐ | Human research participants |
| ☒ | ☐ | Clinical data |
| ☒ | ☐ | Dual use research of concern |

## Methods

| n/a | Involved in the study | |
|---|---|---|
| ☒ | ☐ | ChIP-seq |
| ☒ | ☐ | Flow cytometry |
| ☒ | ☐ | MRI-based neuroimaging |
